# An automated method for precise axon reconstruction from recordings of high-density micro-electrode arrays

**DOI:** 10.1088/1741-2552/ac59a2

**Published:** 2022-03-31

**Authors:** Alessio Paolo Buccino, Xinyue Yuan, Vishalini Emmenegger, Xiaohan Xue, Tobias Gänswein, Andreas Hierlemann

**Affiliations:** Department of Biosystems Science and Engineering, ETH Zurich, Switzerland

## Abstract

**Objective:**

Neurons communicate with each other by sending action potentials through their axons. The velocity of axonal signal propagation describes how fast electrical action potentials can travel. This velocity can be affected in a human brain by several pathologies, including multiple sclerosis, traumatic brain injury and channelopathies. High-density microelectrode arrays (HD-MEAs) provide unprecedented spatio-temporal resolution to extracellularly record neural electrical activity. The high density of the recording electrodes enables to image the activity of individual neurons down to subcellular resolution, which includes the propagation of axonal signals. However, axon reconstruction, to date, mainly relies on manual approaches to select the electrodes and channels that seemingly record the signals along a specific axon, while an automated approach to track multiple axonal branches in extracellular action-potential recordings is still missing.

**Approach:**

In this article, we propose a fully automated approach to reconstruct axons from extracellular electrical-potential landscapes, so-called “electrical footprints” of neurons. After an initial electrode and channel selection, the proposed method first constructs a graph based on the voltage signal amplitudes and latencies. Then, the graph is interrogated to extract possible axonal branches. Finally, the axonal branches are pruned, and axonal action-potential propagation velocities are computed.

**Main results:**

We first validate our method using simulated data from detailed reconstructions of neurons, showing that our approach is capable of accurately reconstructing axonal branches. We then apply the reconstruction algorithm to experimental recordings of HD-MEAs and show that it can be used to determine axonal morphologies and signal-propagation velocities at high throughput.

**Significance:**

We introduce a fully automated method to reconstruct axonal branches and estimate axonal action-potential propagation velocities using HD-MEA recordings. Our method yields highly reliable and reproducible velocity estimations, which constitute an important electrophysiological feature of neuronal preparations.

## 1 Introduction

Axons are assumed to be faithful conductors of action potentials (APs) that encode and transmit information between individual neurons. Traditionally, axons are often considered as simple transmission cables, whose role is the reliable conveyance of APs to the presynaptic terminals of synaptically connected neurons [1]. Owing to recent technology advancements, such reductionist view of the role of the axon is being challenged. A growing body of evidence suggests that axons may provide important contributions to neuronal information processing [2, 3]. For example, the waveform of APs has been shown to be modulated during axonal conduction, which facilitated synaptic transmission to postsynaptic neurons [4]. Moreover, studies using two-photon imaging have found that structural changes of axonal arbors are involved in circuit-level mechanisms of perceptual learning [5]. Therefore, a precise tracking of axonal arbors, including the length of the axonal branches, number of branching points, and AP conduction velocities, will help to shed light onto mechanisms involved in axonal growth during development, axonal-AP modulation and their impact on neuronal signaling.

Due to the small diameters of axons of around 200 nm, the tracking of complete axonal arbors is challenging. Several classical electrophysiological techniques have been used for measurements and detection of AP propagation along axonal arbors. Whole-cell patch clamp, for example, has been used to measure the fidelity of AP propagation using dual patching at the soma and axonal blebs [6] or using cell-attached extracellular recordings in unmyelinated axons [7]. However, due to limitations in simultaneously recording from multiple sites along axons, the patch-clamp technique cannot be used to map axonal arbors. Alternatively, morphological information about neurons, including their axonal arbors, can be obtained with high-resolution imaging techniques. Recent advancements in imaging techniques, such as high-content imaging (HCI) [8, 9], have enhanced spatial resolution of acquired images. Together with the advances in image processing techniques [10, 11, 12], the reliability and throughput of such imaging methods allow for automatic tracing of neurites and their interconnections [12]. Yet, the use of imaging techniques requires fluorescent labels [13, 14], that may alter the physiological properties of the cells [15] through phototoxicity and photobleaching. In addition, it is difficult to extract axon morphologies in high-density cultures from imaging data, where axons form bundles. HCI after post-hoc immunostaining ensures high spatial resolution, but axonal properties can only be investigated in live neurons.

High-density microelectrode arrays (HD-MEAs) have also been used to acquire electrophysiological signals of neurons at high temporal and spatial resolution [16]. Previous studies demonstrated the possibility to extract detailed representations of the extracellular electrical-potential landscape, so called “electrical footprints” of individual neurons from HD-MEA recordings by applying spike sorting and spike-triggered-averaging techniques [17, 18, 19]. These electrical footprints reflect the neurons’ morphology, so that researchers can use them for tracking neurite outgrowths of single neurons. However, the number of axonal arbors that could be extracted in the above-mentioned studies was limited to a few tens of cells in each sample due to tedious manual procedures to select and assign axonal signals. To date, no automatized method for extraction of morphological and functional information from large-scale electrophysiological HD-MEA data is available.

Building upon ideas and concepts of recent previous work [20, 21], we developed a novel, fully automated method to accurately reconstruct axonal arbors from functional electrophysiological HD-MEA recordings. Our method relies on a graph-based approach to reconstruct axonal branches and estimate AP conduction velocities. The proposed automatic method for reconstruction of axonal arbors and determining the corresponding AP conduction velocities from large-scale HD-MEA recordings opens up new possibilities to use axonal properties as electrophysiological biomarkers for studying compound efficacy and neural development as well as for drug screening and disease modeling.

## 2 Methods

In this section, we first introduce the biophysical simulation framework that we used as development test bench and for validation. Next, we describe in detail the implementation of the axonal tracking algorithm. Finally, we describe the protocols and procedures for experimental validation of our method.

### 2.1 Biophysical simulations

In order to develop and validate our axonal tracking approach, we initially used biophysical simulations. A simulation environment allowed us to explore complexities in the extracellular action potentials in a controlled manner, and to refine our method to deal with different cases. The simulations were carried out using LFPy 2.2.1 [22, 23] and NEURON 7.8.2 [24].

#### 2.1.1 Cell morphologies

**Figure 1:**
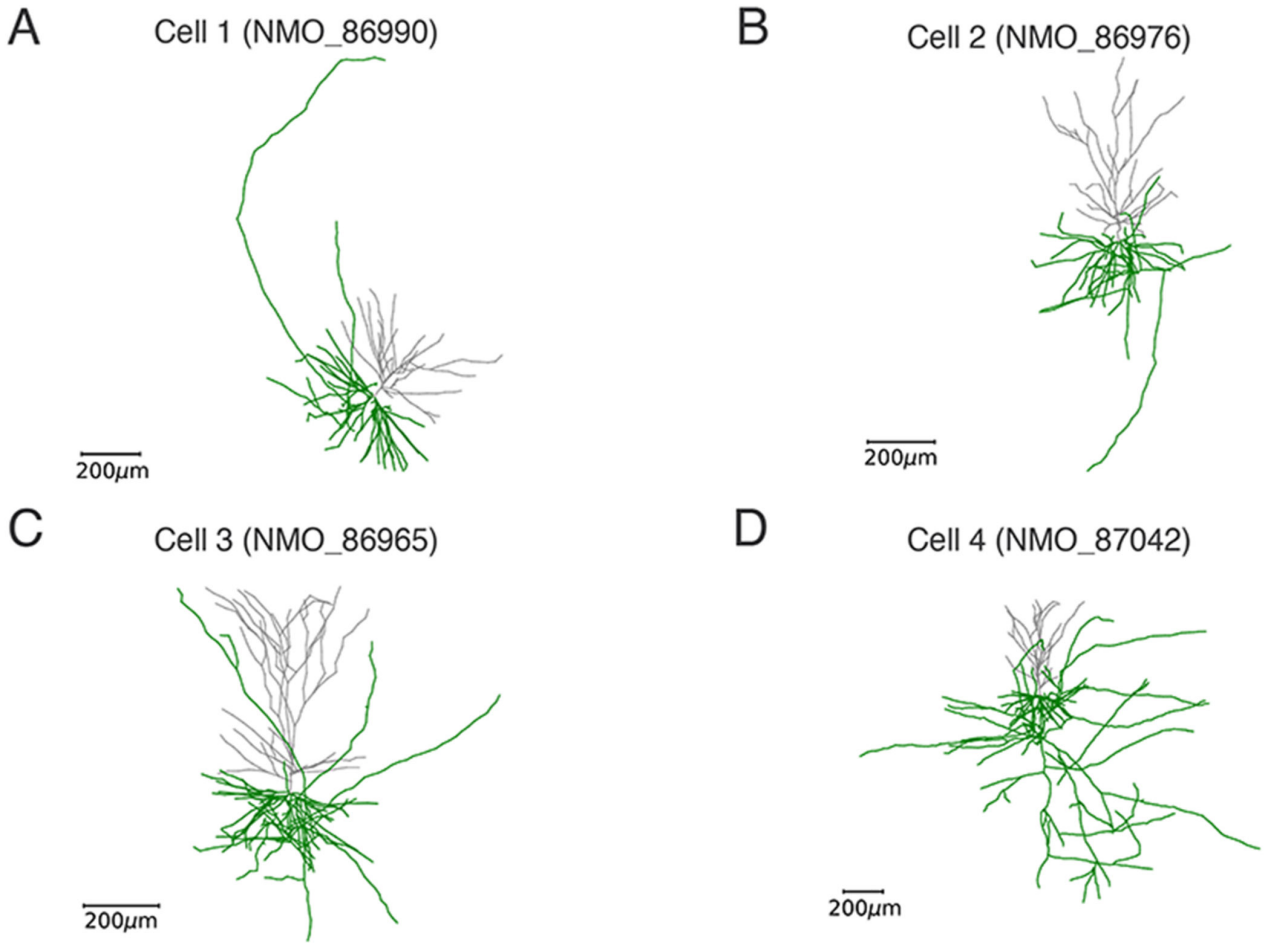
Neuron morphologies for biophysical simulations. Realistic morphologies from the Allen Institute of Brain Science cell-type database: Cell 1 (NMO_86990) - A, Cell 2 (NMO_860976) - B, Cell 3 (NMO_86965) - C, and Cell 4 (NMO_87042) - D. The axonal arbors are colored in green, while dendrites are in grey.

We used morphological reconstructions of human pyramidal neurons from the Allen Institute of Brain Science cell-type database [25]. The cell models were downloaded from the Neuromorpho.org website [26] and included four samples (NeuroMorpho IDs: Cell 1 - NMO_86990 - Figure 1A, Cell 2 - NMO_86976 - Figure 1B, Cell 3 - NMO_86965 - Figure 1C, Cell 4 - NMO_87042 - Figure 1D). Since axonal tracking will be performed for cells cultured on a flat MEA substrate [19], the morphology of which extends principally in two dimensions, we modified the morphologies by setting all z-values to 0 μm, i.e., generated planar morphologies.

#### 2.1.2 Cell biophysics

For all cell models, biophysical properties were added in order to obtain realistic AP generation and axonal AP propagation. The membrane capacitance was set to 1 *μF/cm* ^2^ for all compartments. Dendritic trees were defined to feature only passive membrane properties, with a membrane resistance of 150 *k*Ω and a reversal potential of −85 *mV*. The somatic compartments featured sodium and potassium Kv1 channels [27], with maximum conductances of 500 and 100 *S/cm* ^2^, respectively. The axonal tracts also featured sodium- and potassium-channel conduction mechanisms, with maximum conductances of 500 and 400 *S/cm* ^2^, respectively. The reversal potential for the sodium channel was set to 55 *mV*, and for the Kv1 channel to −98 *mV* [27]. The axial resistance was set to 80 Ω*cm* and the temperature to 33° Celsius. The resting potential was set to −85 *mV*, and we simulated the cell model for 100 ms. The time step for the simulations was set to 0.03125 *ms*, yielding a sampling frequency of 32 *kHz*. In order to induce a single AP, we stimulated the cell with two to five consecutive synaptic inputs (ExpSyn mechanism - 1 ms between inputs) directly to the soma of the neuron.

#### 2.1.3 Modeling of extracellular signals

Extracellular potentials were modeled with a well-established forward-modeling scheme using the LFPy software [23]. Assuming a quasi-static, linear, isotropic, homogeneous, and infinite medium, the contribution of a neuronal transmembrane current *I_i_* (*t*), distributed over a line source (*line-source model*), centered at a point ***r**_i_* to the potential *φ_i_* (***r**_j_*, *t*), measured by an electrode at position ***r**_j_*, can be computed as:

(1) $$\phi_{i}\left({\text{r}}_{j},t\right)=\frac{1}{4\pi \sigma }I_{i}(t)\int^{\text{​}}\frac{\text{d}r_{i}}{∥{\text{r}}_{j}-{\text{r}}_{i}∥}.$$

where *σ* is the extracellular conductivity (0.3 *S/m*). While the assumption of an infinite and homogeneous milieu is clearly violated in the presence of a highly insulating HD-MEA surface [28, 29], we did not apply any correction, e.g., by using the method of images [28]. A correction would only change the signal amplitudes but not alter signal timing and the relative signal amplitude distribution across the electrodes, which are pivotal for applying the proposed tracking algorithm.

The HD-MEA device was simulated using the MEAutility package [30], which is integrated in LFPy (version ≥ 2.1). A 100x100 electrode grid (10’000 electrodes in total) featuring a pitch of 17.5μm, which represents the state-of-the-art of HD-MEA devices [31, 32, 33], was placed on the x-y plane at a vertical distance of 10 μm below the neuronal-cell plane. To represent the spatial extension of the electrodes, they were modeled as squares with a 5 μm side length. The *recorded* electrical potential was computed as the average over 10 points randomly positioned within the electrode surface using the so-called *disk-approximation* [22].

**Figure 2:**
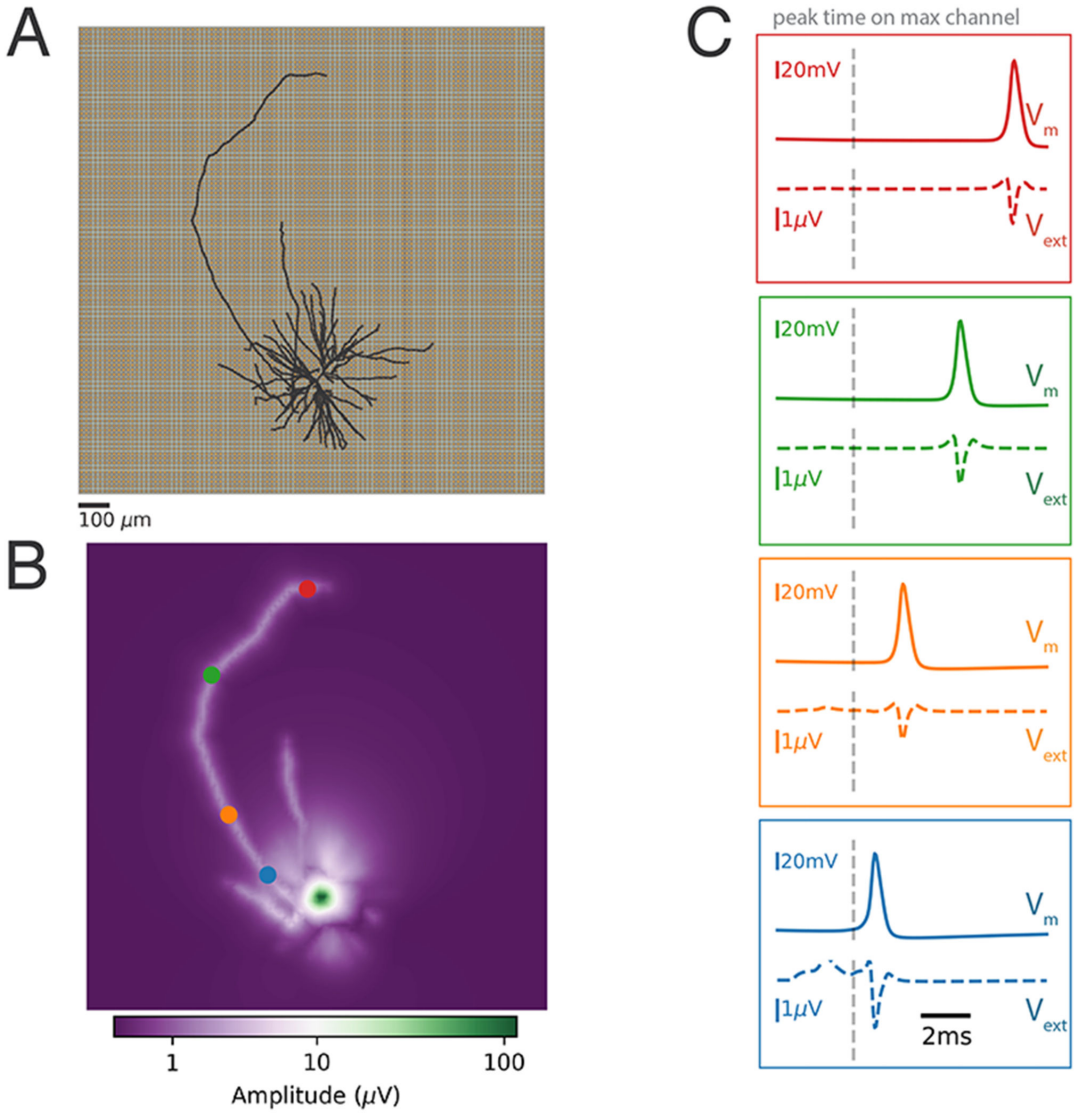
Simulation of extracellular signals. **A)** Representation of the HD-MEA and the *Cell 1* neuron located on top of the MEA. **B)** Amplitude map (in log scale) of the extracellular action potentials. Several axonal branches are clearly visible. **C)** Membrane potentials (*V_m_* - top) and extracellular signals (*V_ext_* - bottom) for the four points, indicated in color, along the longest axon in panel B. The vertical grey dotted line indicates the time of occurrence of the signal peak on the electrode featuring the largest AP amplitude.

Figure 2A shows a visualization of the Cell 1 (NMO_86990) in black on top of the electrode grid of the HD-MEA. Displayed is the complete morphology including axons and dendrites. The extracellular electrical-potential amplitude map, referred to as the *footprint* (channel-wise peak-to-peak amplitude on a log scale), is shown in Figure 2B, while the insets of Figure 2C, display the aligned intracellular and extracellular signals, showing the axonal propagation from the proximal part of the longest axon (blue, bottom) to the distal end (red, top).

### 2.2 Graph-based algorithm

In this section, we describe the proposed algorithm, the application of which includes four main steps: *i)* channel selection, *ii)* graph construction, *iii)* axonal-branch reconstruction, and *iv)* axonal-arbor pruning and velocity estimation. The method originated from previous ideas and concepts [20, 34], which have been organized, modified, validated, and assembled to obtain a coherent and fully functional method for axonal-arbor reconstruction. In Section 4 we compare the presented new method to the previously used approaches.

The method assumes that the raw extracellular data have been spike-sorted and that the *templates*, i.e., the average extracellular waveforms have been computed for each individual unit. Each unit and its extracellular template are analyzed separately by the algorithm.

#### 2.2.1 Channel selection

**Figure 3:**
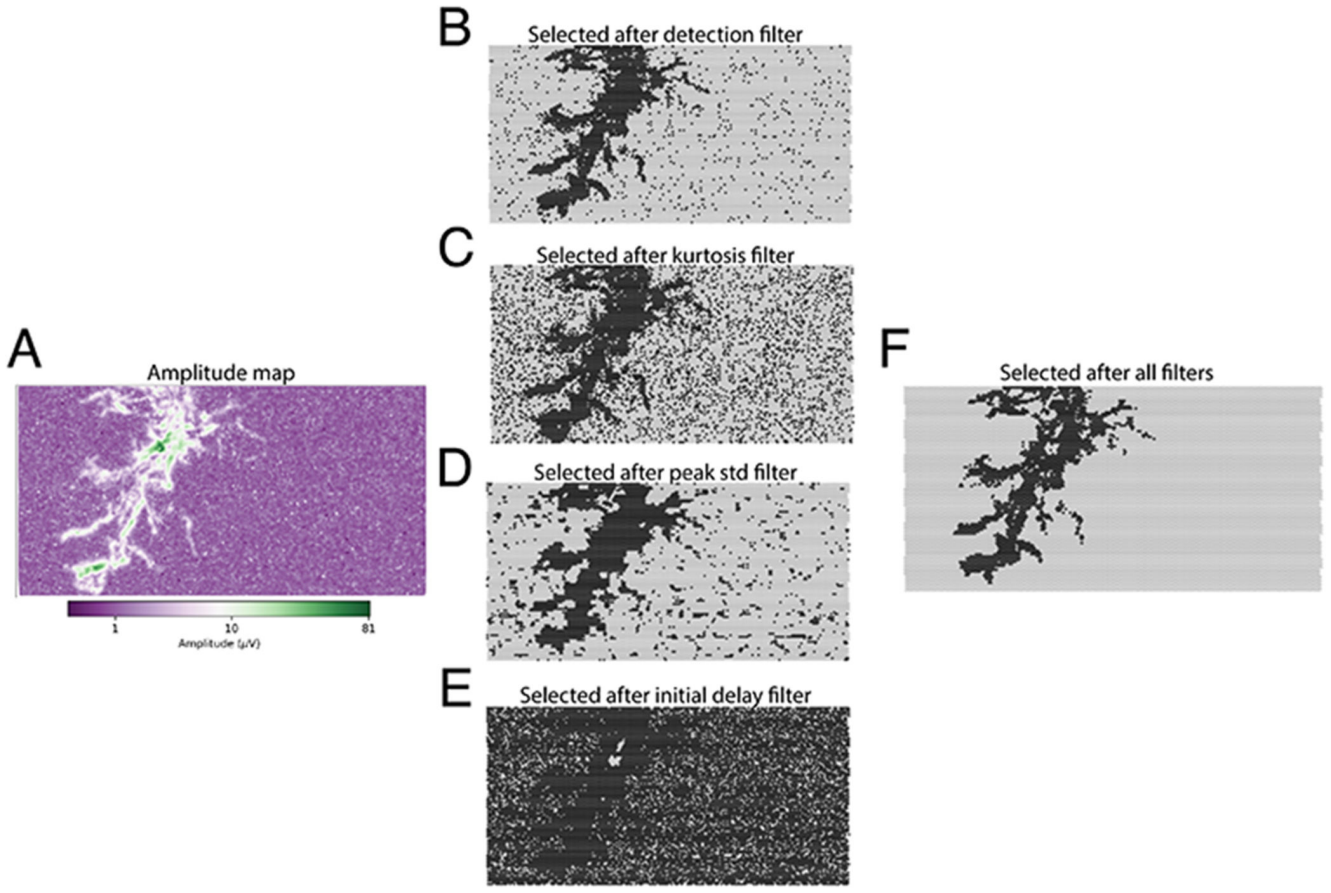
Channel selection procedure. **A)** Amplitude map (log scale) of a neuron footprint. **B-E)** Selected (black) and excluded (grey) channels after filtering for amplitude (B), kurtosis (C), peak time standard deviation (D) and initial delay (E). Selected (black) channels after combining all filters.

In order to track axonal branches, first, a subset of electrodes/channels needs to be selected that can be used for axonal tracking. Four *filters*, based on signal amplitudes, kurtosis, peak time standard deviations, and initial signal delays are available. An appropriate channel selection depends on many factors, such as the probe geometry and the noise level; therefore, the proposed method gives freedom to the user to modify the filter configuration to maximize tracking performance. In the following section, we briefly describe how the different filters operate and we display, in Figure 3, the channel selection for a real neuronal footprint, which was obtained in an HD-MEA recording using ∼20’000 channels [20]). The footprint is shown in Figure 3A, the channel selection for each available filter in Figure 3B-E and the selection using all 4 filters in Figure 3F.

Amplitude filter The first available filter is a *detection filter* based on the amplitude of the recorded signal. Only channels with a peak-to-peak amplitude larger than a detection threshold are kept for further processing. The detection threshold can be defined relative to the largest signal amplitude of the recording (default) or as an absolute value in μV, and the default setting is 0.01 (1 %). Figure 3B shows all available channels in grey and the selected channels after applying the detection filter in black.

##### Kurtosis filter

Second, a filter based on kurtosis can be used in order to ignore channels that may contain only noise. A noisy channel, in fact, may pass the detection filter unnoticed. However, if a channel features *signal spikes*, its kurtosis should be above zero, i.e., it should exhibit a supergaussian distribution. The default setting of the kurtosis filter is 0.3, and all channels with a kurtosis value below this threshold are removed. Figure 3C shows all available channels in grey, and the selected channels after application of the kurtosis filter in black.

##### Peak time standard deviation filter

A third available filter relies on the standard deviation of the occurrence time of the signal peaks. After computing the peak time for each channel (the time at which the maximum negative signal occurs), we compute the standard deviation of these values over neighboring channels (channels within 30 μm distance are selected per default) [18]. A low standard deviation indicates that the peak times of neighboring channels are coherent, i.e., they carry signal. Conversely, a large peak time standard deviation suggests that channels mostly contain noise, which renders the peak time more random. The recommended threshold for this filter is 1 ms, and the channel selection based on this filter is shown in Figure 3D.

##### Initial delay filter

Since our aim is to track axons, the fourth and final available filter is targeted at finding channels with an axonal signal. Assuming the largest-amplitude channel (termed *initial channel*) being in proximity to the axon initial segment [35], the filter removes all channels whose signal peaks occur before the *initial channel* peak time plus an additional delay (set to 0.1 ms by default). This removal is done to *wait* until electrical-signal propagation has entered the axonal branches. Figure 3E shows all available channels in grey, and the selected channels after using the initial-delay filter in black.

All selection filters are applied separately, and the final channels selected correspond to the intersection of the channels selected by each individual filter (Figure 3F). Finally, isolated channels (selected channels without a *neighbor* within 100 μm distance) are removed from the selection.

#### 2.2.2 Construction of the graph

After the channel selection, the *remaining* channels are used as the nodes of a graph. Prior to the graph construction, however, the selected channels are sorted based on the following heuristic:

(2) $${\text{h}}_{\text{init}}=\alpha_{init}\cdot a_{\text{n}}+\left(1-\alpha_{init}\right)\cdot {\text{p}}_{\text{n}}$$

where *a_n_* are the normalized amplitude values, ***p_n_*** are the normalized peak latencies of the selected channels, and *α_init_* is a scalar that weighs the contributions of amplitude and peak latency. The normalization step is performed to be able to combine two very different units (μm for amplitudes and ms for peak latencies). The channel sorting will influence the order of initial channels chosen to construct axonal branches. By default, *α_init_* is set to 0.2, so that the channels with signal-amplitude peaks that occur comparably late are favored, and among those, the channels featuring the largest amplitudes.

When selected channels are sorted, they are used as *nodes* to populate a directed graph. The graph is built using the NetworkX Python package [36]. Next, *edges* are added to the graph. For each node, at most n_neighbors edges (default: 3) can connect to other candidate nodes if: *i)* the candidate node has a signal peak occurring earlier in time, and *ii)* the candidate node is within a maximum distance (default: 100 μm). Among the candidate nodes that satisfy these two requirements (there can be more than n_neighbors depending on the electrode density of the MEA), the channels featuring the largest amplitudes and the lowest distances are favored. Channels for which there is no other channel with an earlier peak occurrence (excluding the initial channel) are connected to the initial channel if they are within a defined spatial range (default: 200 μm distance). Each edge is added to the graph and it is weighted by the average amplitude of its parent and child nodes (edge amplitude). After all edges have been added to the graph, all edge amplitudes are retrieved and normalized between 0 and 1. Then, their values are reversed so that the largest amplitude has a value of 0, and the smallest one is assigned a value of 1. We denote these normalized edge amplitudes as ***h_edge_***, since they are used as a heuristic to find axonal branches. For all edges connecting to the initial channel, the *h_edge_* value is set to 2.

**Figure 4:**
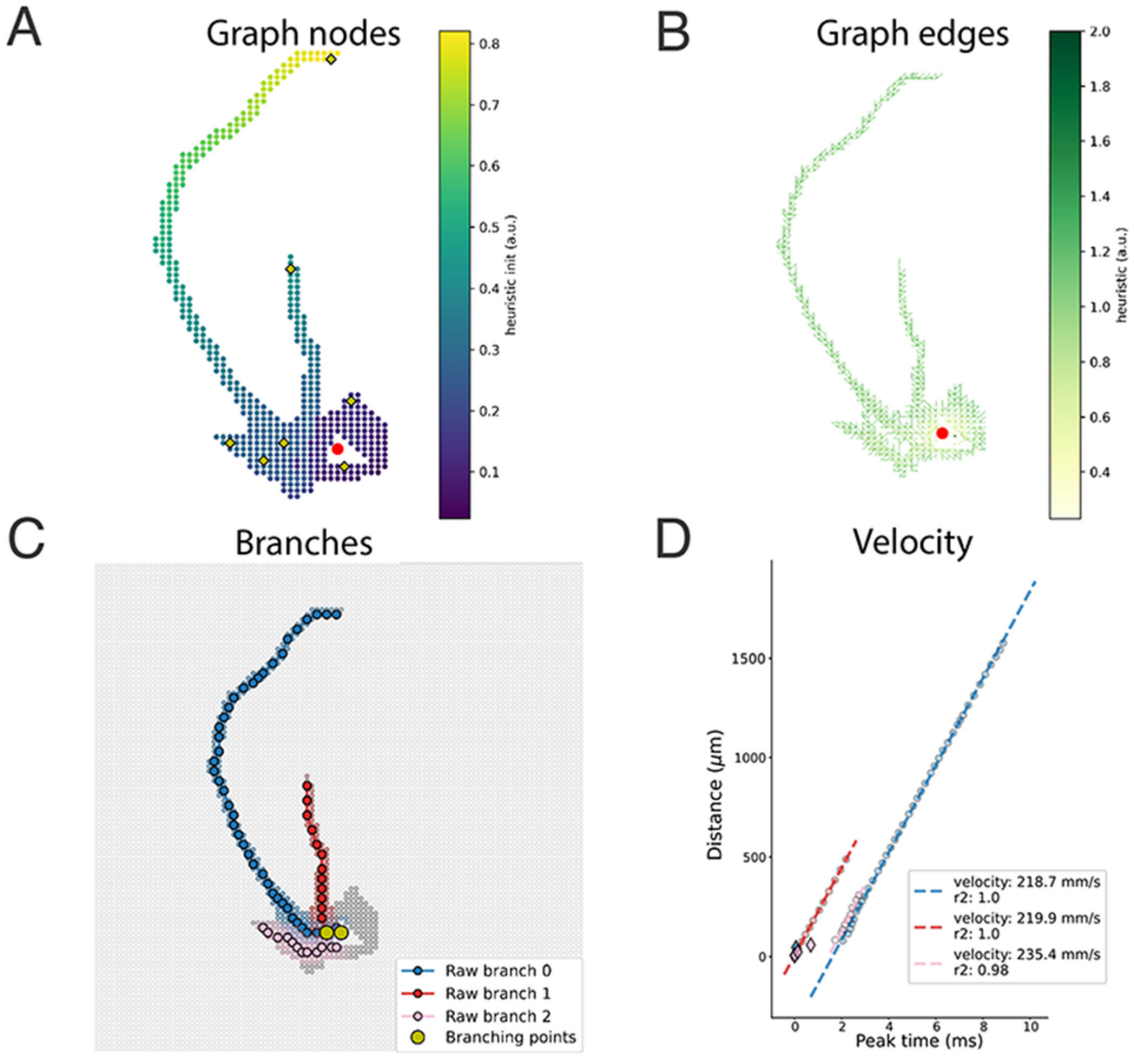
Axonal reconstruction method. **A)** Graph nodes colored according to *h_init_* values for the *Cell 1* neuron. The nodes marked with a yellow diamond indicate nodes for which a path towards the initial channel has been searched for. **B)** Graph edges colored according to *h_edge_* values. **C)** Identified raw axonal paths. The dark grey dots are the selected channels. The colored nodes around an identified path are the neighbor nodes for that path, which have been removed for further searches. The yellow circles indicate the branching points. **D)** Robust velocity estimation. For each reconstructed branch, a robust estimator was used to fit the axonal AP propagation velocity. The blue and pink diamonds at the bottom left show detected outliers from branch 0 (blue) and branch 2 (pink) respectively, which were removed from the cleaned paths.

The graph nodes for the model of *Cell 1* are shown in Figure 4A. The nodes are colored according to ***h_init_*** values. Figure 4B shows the edges colored according to ***h_edge_*** values for the same cell model. It becomes evident that the ***h_init_*** values exhibit local maxima at the axon ends and that lower values of ***h_edge_*** nicely coincide with axonal paths.

#### 2.2.3 Axonal branch reconstruction

The two heuristic functions (***h_init_*** and ***h_edge_***) are used to reconstruct axonal branches. The goal of this step is to find possible paths that coincide with axonal branches. Since graph nodes are already sorted by *h_init_*, this procedure loops through the nodes and attempts to find paths ***P*** towards the initial channel while minimizing the edge heuristic ***h_edge_***. A path is searched between a node and the initial channel only if the node is a local maximum in the ***h_init_*** space, i.e., it has the largest value of *h_init_* compared to other channels within a fixed distance (default: 100 μm). This approach ensures that only a small number of paths is reconstructed and improves the efficiency of the method. The nodes indicated as yellow diamonds in Figure 4A represent the local maxima that have been identified as starting nodes for axonal branches.

For each starting node, the shortest path is obtained using the *A* ^*^ method [37], which is an optimal-path-search algorithm to find the shortest path in a graph by minimizing a cost function (we used the astar_path() function of the NetworkX Python package). The method can also consider a distance function between nodes, that we defined as:

(3) $${\text{d}}_{\text{n}}={\left[\frac{\text{d}-min(\text{d})}{max(\text{d})-min(\text{d})}\right]}^{e}$$

where *d* is the distance between two nodes that are connected by an edge and *e* is the configurable exponential (2 by default). The value of the exponent *e* is chosen to be larger than 1 to minimize *long jumps* between nodes, as large distances will be more penalized in the super-linear space. To summarize, the *A* ^*^ method finds the path that minimizes:

(4) $$\underset{\text{P}}{\text{argmin}}\underset{p\in \text{P}}{\sum }\left(h_{edge_{p}}+d_{n_{p}}\right)$$

where *p* is a single node, and ***P*** is the set of nodes that makes up a path.

When a path is found, the channels within the neighborhood of each channel in the path (by default, within 100 μm) are appended to the *neighbor channels set*. If a newly identified path includes channels that are already in the *neighbor channels set* (i.e., it is neighboring an already existing path), all new channels in this *neighbor channel set* are removed from the new path. The channel with the earliest peak time in the new path is then connected to the closest node of the already identified closest path. In this case, the respective node becomes a *branching point*. After all paths and all branching points have been found, paths are pruned and merged. A path is pruned if a portion of it extending from a branching point does not have at least three points by default (this value is adjustable by the user). Finally, pairs of paths that, after pruning, share a branching point which corresponds to the last channel of one path and the first channel of the other path are merged. After pruning and merging, a path is stored as a *raw* axonal branch if two conditions are met: *i)* the length of the path is larger than a path length threshold (default: 100 μm), and *ii)* the path contains at least a minimum number of points (default: 5). Once a path has been accepted, all the channels of the path and the ones within a *neighbor radius* (default: 50 μm) of any of its nodes are stored in the memory and excluded from further searches. This step ensures that no duplicate paths are found for the same axonal branch.

The identified branches for the *Cell 1* model are shown in Figure 4C. In this case, three raw branches were found (blue, red, pink). The grey dots are the selected channels and the shaded nodes around each path (with the same color) indicate the channel neighbors, which were removed from further path searches. The yellow circles represent the branching points.

The full algorithm to estimate raw branches is described in Algorithm 1 in Appendix A.

#### 2.2.4 Path cleaning and velocity estimation

After obtaining the set of paths, axonal velocities can be estimated. Peak times are computed as the difference between the occurrence time of the signal peak at each node and the peak time occurrence of the first node in the path (which is the one featuring the earliest signal peak by definition). Cumulative distances are calculated by integrating the distances between the channels along the path.

Once peak times and distances have been computed, a robust linear fit using the Theil-Sen regressor (using scikit-learn [38]) is used to reduce contributions of possible outliers. The velocity estimate is derived from the slope of the regression line. We use a non-parametric and robust approach to identify and remove possible outliers from the path. We first compute the prediction error for each channel. We then identify outliers as nodes with a prediction error above *N* times the median absolute deviation (MAD) of the error distribution (*N* is 8 by default) and above a fixed threshold (30 μm by default). Outliers are then removed from the axonal branches, and a new linear fit is computed.

In some cases, it could happen that a path presents a *shortcut* either between different branches or within the same branch with an undetected axonal section. In this case, a jump in the peak times is observed. In order to correct for this unwanted behavior, the method attempts to split the path when jumps in the peak times are detected (>1 ms by default), and to fit the splitted sub-paths separately. If the average *R* ^2^ of the sub-paths is larger than the *R* ^2^ of the original path, the path is split and the sub-paths are considered as separate branches. Finally, axonal branches with an *R* ^2^ value below a user-defined threshold (default 0.9) are discarded.

Figure 4D shows the peak latencies (x-axis), cumulative distances (y-axis), and the fitted AP propagation velocities (dashed lines) for the raw branches displayed in Figure 4C for the *Cell 1* model. The linear fit achieves a very high *R* ^2^ value, partially owing to the removal of outliers of the blue and pink branches, depicted as diamond shapes.

### 2.3 Software implementation and code availability

The implementation of the above-described algorithm is available as an open-source Python package called axon_velocity on GitHub ( https://github.com/alejoe91/axon_velocity ) and on PyPi ( https://pypi.org/project/axon-velocity/ ). All the code needed to reproduce figures in this article can be found in the figure_notebooks folder of the GitHub repo, while the required data are available at Zenodo ( https://doi.org/10.5281/zenodo.4896745 ).

The graph-based algorithm takes the electrode array template (a numpy array with dimensions num_channels x num_samples), the x-y electrode locations (a numpy array with dimensions num_channels x 2), and the sampling frequency as required arguments. Additionally, all algorithm-specific parameters can be passed as extra arguments:



                  import
                  axon_velocity
                  as
                  av


params = av.get_default_graph_velocity_params()


gtr = av.compute_graph_propagation_velocity(template, locations, sampling_frequency, **params)


The returned gtr object is a GraphAxonTracing object, which contains the following fields:

- branches: list of dictionaries for the detected axonal branches. Each dictionary contains the following fields.
  - channels: selected channels in the path
  - velocity: velocity estimate in *mm/s*
  - offset: offset (intercept) of velocity estimate
  - r2: *r* ^2^ of the AP velocity fit
  - error: standard error of the linear fit
  - pval: p-value of the linear fit
  - distances: array with cumulative distances computed along the branch
  - peak_times: array with signal peak occurrence time differences to initial channel
- selected_channels: list of selected channels used for axonal tracking
- graph: the NetworkX graph used to find axonal branches

In Table 2 of Appendix B we list and describe the additional parameters (**params), their default values, their types, and a brief description of their role.

### 2.4 Experimental procedures

#### Ethics statement

All animal experimental protocols were approved by the Basel-Stadt veterinary office (cantonal no. 2358) according to Swiss federal laws (national no. 30692) on animal welfare and were carried out in accordance with the approved guidelines.

#### High-density microelectrode arrays

To validate the tracking algorithm with experimental recordings, we used data from two types of HD-MEA chips: the first device features 26’400 electrodes with a center-to-center electrode distance of 17.5 μm and can record from up to 1024 channels simultaneously at 20 kHz [31, 32] (referred to as MEA1k); the second device is a dual-mode HD-MEA including switch-matrix and active-pixel readout schemes for electrodes [39, 20] (referred to as DualMode). It features a full-frame readout of 19,594 electrodes at a sampling rate of 11.6 kHz; the center-to-center electrode distance is 18μm.

#### Cell cultures and plating

Rat primary neurons were obtained from dissociated cortices of Wistar rats at embryonic day 18, using the protocol described in Ronchi et al. [21].

Prior to cell plating, HD-MEA chips were sterilized using 70% ethanol for 30 minutes. Ethanol was then removed, and the chips were rinsed three times with sterile tissue-culture-grade water. The HD-MEA chips were coated with a layer of 0.05% polyethylenimine (Sigma) in borate buffer to render the surface more hydrophilic. On the plating day, a layer of laminin (Sigma, 0.02 mg/mL) in Neurobasal medium (Thermo Fisher Scientific) was added on the array and incubated for 30 minutes at 37 °C to promote cell adhesion. We dissociated cortices of E-18 Wistar rat enzymatically in trypsin with 0.25% EDTA (Gibco), followed by trituration. Cell suspensions of 15,000 to 20,000 cells in 7 *μ*L were then seeded on top of the electrode arrays. The plated chips were incubated at 37 °C for 30 min before adding 2 mL of plating medium. The plating medium consisted of Neurobasal, supplemented with 10% horse serum (HyClone, Thermo Fisher Scientific), 0.5 mM Glutamax (Invitrogen), and 2% B-27 (Invitrogen). After 3 days, 50% of the plating medium were replaced by a growth medium, which consisted of D-MEM (Invitrogen), supplemented with 10% horse serum, 2% B27, and 0.5 mM Glutamax. The procedure was repeated twice a week. The chips were kept inside an incubator at 37°C and 5% CO2. All experiments were conducted between days in vitro (DIVs) 10 and 28.

#### Extracellular recordings and analysis

For the MEA1k system, only 1024 channels of the array’s 26’400 electrodes can be recorded simultaneously, therefore an *axon scan* assay was performed: we sequentially recorded 33 different configurations of randomly placed electrodes in order to cover the entire chip area, while the 200 electrodes showing the highest spontaneous activity were fixed. Each configuration was recorded for 120 seconds. The recorded data were analyzed using SpikeInterface [40]: the signals from the fixed electrodes were concatenated in time and spike-sorted using Kilosort2 [41]. The spike sorting output was automatically curated by removing units with a firing rate lower than 0.1 Hz, an ISI violation threshold [42] higher than 0.3, and a signal-to-noise ratio lower than 5. Afterwards, the automatically curated data was exported to Phy [43, 44] for visual inspection and manual curation. The manually curated data were then used to extract full templates across the entire array: first, the spike trains were categorized depending on the start and end time of the different configurations; second, the template for each configuration was computed as the median of all extracted waveforms; finally, templates extracted from different configurations were averaged to obtain the final full template.

For the DualMode system, we analyzed a short full-frame recording of *∼* 285 seconds. As most spike sorters do not handle more than *∼* 1000 channels, we first computed the spike rate of each channel using a spike detection based on 5 times the median absolute deviation. We then selected the 1024 most active channels and spike sorted them using Kilosort2 and the same automatic curation as for the MEA1k recordings. A final manual curation step using Phy was performed, and templates were extracted by combining spike times and the full-frame recording.

After spike sorting and the computation of templates for each sorted unit, the proposed axon-reconstruction method was applied to the templates of each sorted unit separately.

### 2.5 Evaluation of the tracking performance

In order to evaluate the performance of the proposed axon-tracking algorithm, we used the simulated data as ground truth. The ground-truth branches of the cell models were matched to the estimated axonal branches using a many-to-one strategy (since the estimated branch could span over one or more ground-truth branches). The matching was performed by computing the median distance of each ground-truth path to each estimated path. We considered possible matches if the median distance between the ground-truth and the estimated paths was below 40 μm. Among the ground-truth branches matched to the same estimated branch, overlapping ground-truth branches were discarded. Overlapping branches were defined as ground-truth branches with more than 20% of their segments being located within a distance of 15μm. In case overlapping ground-truth branches were found, the shortest ones were removed.

After the matching procedure, tracking errors and AP propagation velocities were computed for each estimated branch. The tracking errors were computed as the distance between each channel of an estimated branch and the closest segment of the matched ground-truth branches. Tracking errors were reported as mean±standard deviation in Table 1. In case of velocities, we also computed the absolute velocity error (*abs*(*v_gt_ − v_est_*)) and the relative velocity error (*abs*(*v_gt_ − v_est_*)*/v_gt_*). Here *v_gt_* is the ground-truth velocity – computed as the weighted average of the branch AP propagation velocity with respect to the branch length – and *v_est_* is the estimated branch AP propagation velocity.

## 3 Results

### 3.1 Algorithm performance on realistic, simulated morphologies

In order to validate and assess the performance of the proposed method, we analyzed the axonal reconstructions and velocity estimations of simulated extracellular APs using the realistic morphologies from the Allen Institute database (Figure 1). Already from the morphologies, one can appreciate that the first three neuronal models (Cell 1, Cell 2, Cell 3) displayed well separated axonal branches, while Cell 4 (Figure 1D) showed a much more intricate axonal arborization.

**Figure 5:**
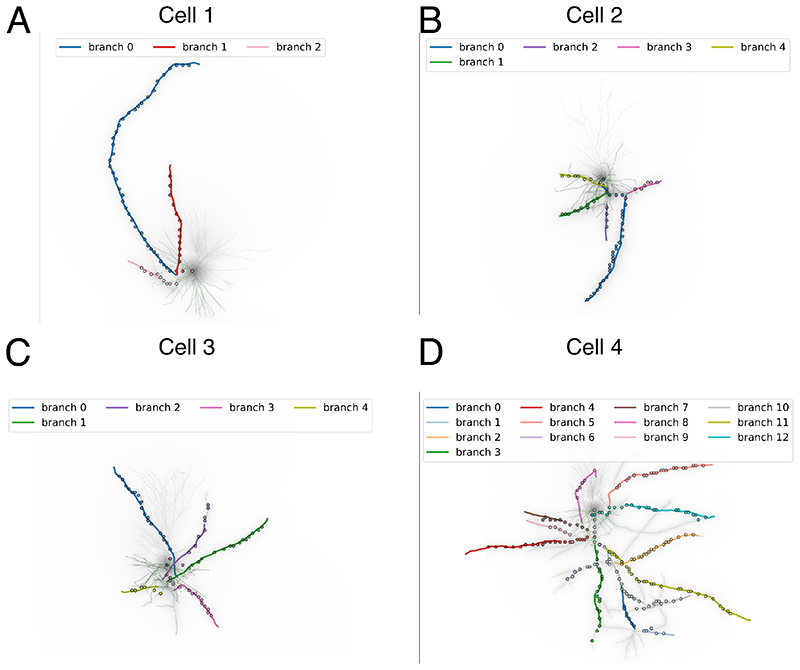
Axonal reconstruction on realistic neuron morphologies. Morphological reconstructions of Cell 1 **A)**, Cell 2 **B)**, Cell3 **C)** and Cell 4 **D)**. Colored lines display ground-truth branches that have been matched to the reconstructed branches (colored circles). The morphology of the cell is shown in the background.

We ran the graph-based algorithm with default parameters (listed in Table 2) and evaluated the tracking results against ground-truth information of the model cells. Figure 5 shows the estimated branches as dots and the matched ground-truth branches as lines. The estimated and corresponding matched ground-truth branches are plotted in the same color. Qualitatively, the developed method correctly identifies the main axonal branches of all tested model cells and shows good performance even for Cell 4, despite the multitude of axonal branches crossing each other. Table 1 shows the ground-truth and estimated velocity, the absolute and relative velocity errors, and the mean and standard deviation of the tracking errors for all estimated branches of the four model cells. In most cases (19 out of 26 axonal branches) the relative error is below 10 %. Higher velocity and tracking errors can be due to a partial match to the ground-truth branch (e.g., branch 0 in Figure 5B and branch 3 in Figure 5C). Nevertheless, the proposed tracking algorithm is capable of correctly reconstructing large portions of the axonal arborization of all model cells.

**Figure 6:**
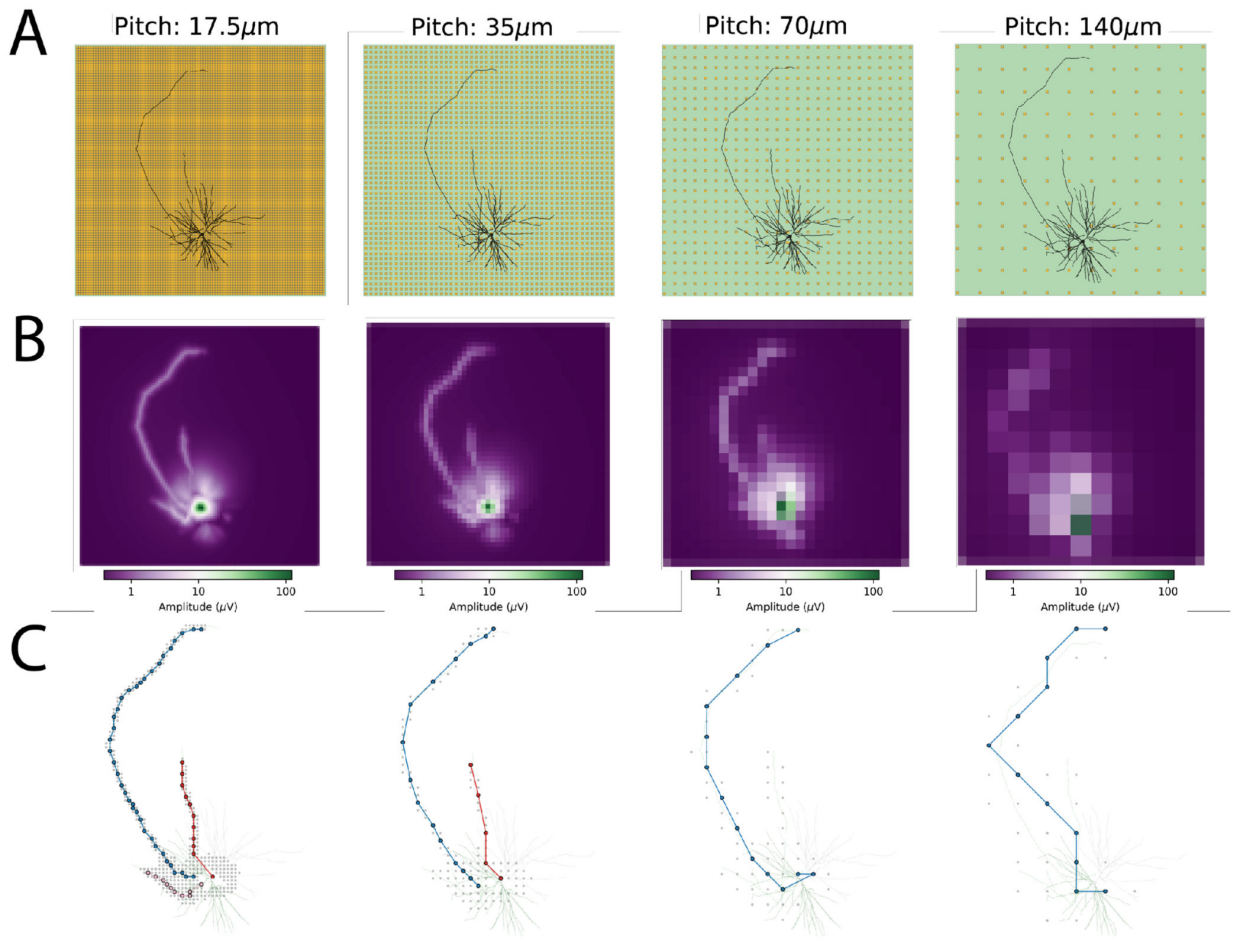
Effect of different MEA electrode pitches on the axon reconstruction performance. Each column represents a different pitch, increasing from left to right. (A) Neuron morphology of Cell 1 on top of the MEAs. (B) Amplitude map of the extracellular signal. (C) Reconstructed branches.

We also looked at the effect of different MEA spatial resolutions on the axonal reconstruction. To do so, we simulated Cell 1 on different MEA models, with increasing pitches of 17.5μm, 35μm, 70μm, and 140μm (keeping the same electrode size of 8 μm). Figure 6 show the results of the axonal reconstruction. Panel A shows the neuron morphology on top of the respective MEA, panel B the amplitude maps, and panel C the algorithm reconstruction (in this case we only changed the neighbor_radius parameter to 2 times the pitch). From panel B, it is evident how the larger pitch affects the electrical image of the neuron, which is reflected in the capability of the reconstruction algorithm to find axonal branches. Already at a pitch of 35 μm, the pink axon branch at the bottom of the cell cannot be traced, while only the main blue branch is found upon further increasing the pitch, which can be only roughly reconstructed in the case of the 140 μm pitch.

### 3.2 Application to HD-MEA recordings

**Figure 7:**
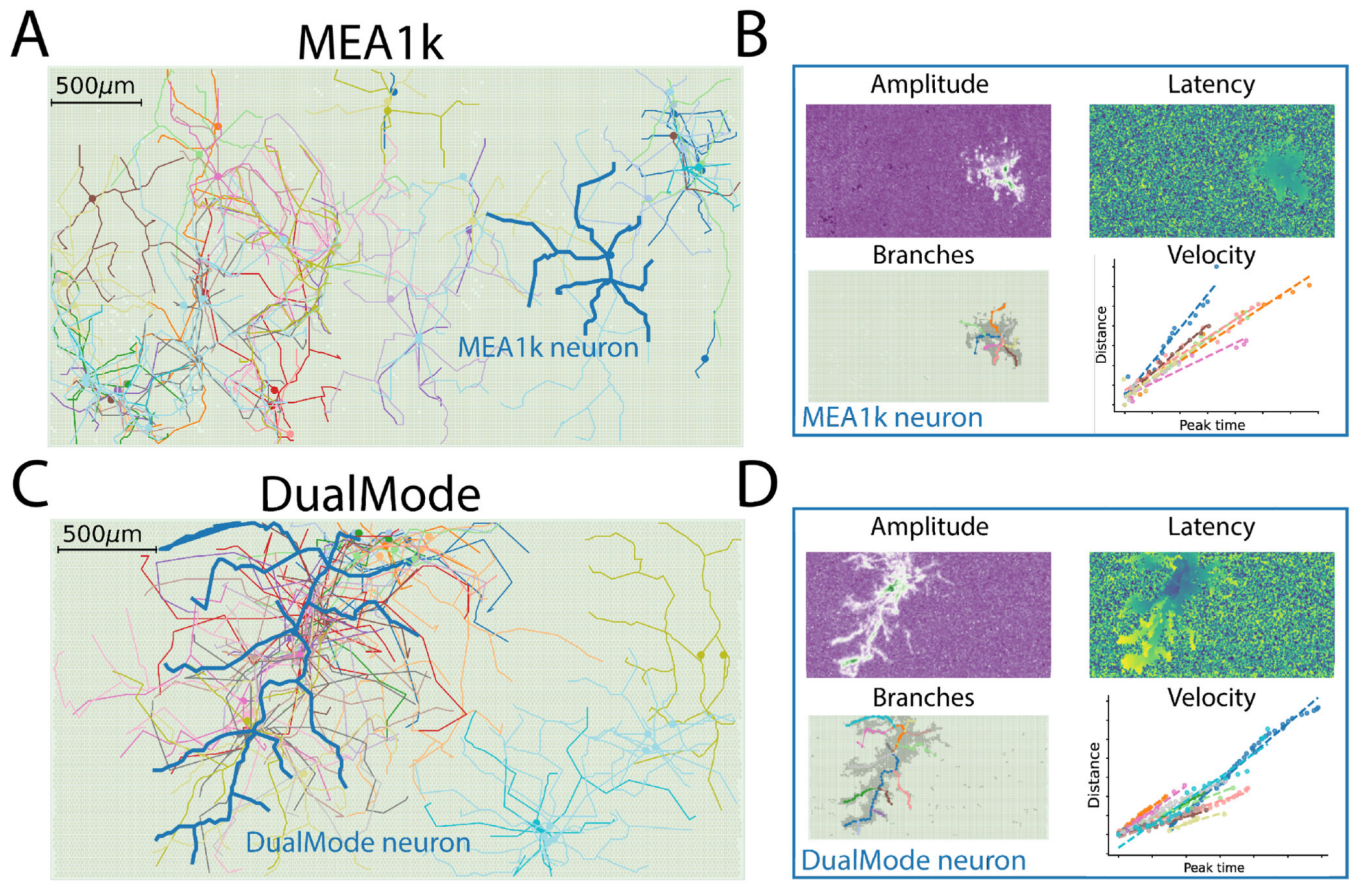
Application to HD-MEA recordings. **A)** Axonal arbors on a MEA1k device. All 67 units with detectable axon branches are displayed with a representation of the MEA (26’400 channels) in the background. **B)** Amplitude map (top left), peak latency map (top right – blue: 0 ms, yellow: 6 ms), reconstructed branches (bottom left), and velocity fits (bottom right) of the “MEA1k neuron” shown in blue in panel A. **C)** Axonal arbors on a DualMode device (51 units with detectable axon branches). **D)** Amplitude map (top left), peak latency map (top right – blue: 0 ms, yellow: 4 ms)), reconstructed branches (bottom left), and velocity fits (bottom right) of the “DualMode neuron” shown in blue in panel C.

After validating the tracking performance of the proposed algorithm on simulated data, we analyzed experimental data from recording sessions, of two different HD-MEAs, a MEA1k and a DualMode recording. In both cases, we ran the proposed tracking algorithm using a detection threshold of 1%, a kurtosis threshold of 0.1, a standard deviation threshold of the signal peak occurrence time of 0.8 ms, and an initial delay of 0.2ms. For the MEA1k dataset, the spike-sorting procedure after manual curation yielded 77 isolated units. Out of these, 67 units had detectable axonal branches. The algorithm found a total of 249 axonal branches, with velocities of 386.03 ± 250.7 *mm/s*, path lengths of 458. 07 ± 257. 03 μm and *R* ^2^ values of 0.94 ± 0. 05. In Figure 7A we show all reconstructed axonal branches with a visualization of the MEA1k device with 26’400 electrodes in the background. Figure 7B shows a representative neuron of Figure 7A (marked in blue). The amplitude map of the template (top left), the peak latency map (top right), the reconstructed branches (bottom left), and the fitted velocities (bottom right) are shown. For this neuron, the channel selection yielded 1252 channels, featuring 8 axonal branches with path lengths of 486.77± 151.15μm, AP propagation velocities of 417.57 ± 116.65 *mm/s*, peak-to-peak extracellular amplitude of 69μV, and *R* ^2^ values of 0.93 ± 0.05.

In the DualMode recording, we found 58 units after spike-sorting and curation. Out of these, 51 had detectable axonal branches (shown in Figure 7C), and a total of 191 branches have been found (velocities: 368.88 ± 203.33 *mm/s*, path lengths: 504.18 ± 317.15 μm, *R* ^2^ values: 0.95 ± 0.05). Similar to Figure 7B for the MEA1k neuron, Figure 7D shows detailed plots for one representative neuron displayed in blue in Figure 7C. For this unit, the channel selection yielded 2819 channels, where 14 axonal branches were traced featuring path lengths of 627.8 ± 426.24 μm, AP propagation velocities of 448.53 ± 173.33 *mm/s*, peak-to-peak extracellular amplitude of 103.2 μV, and *R* ^2^ values of 0.96 ± 0.03.

We showed that the application of the proposed axonal reconstruction algorithm to spike-sorted data of HD-MEAs yields a high-throughput detection and assessment of axonal properties. The algorithm can potentially provide valuable information on axonal properties under physiological and pathological conditions.

## 4 Discussion

In this article, we introduce a novel, fully automated algorithm for reconstruction and AP-propagation velocity estimation of axons using HD-MEAs. The algorithm uses an efficient graph-based approach to reconstruct multiple axonal branches from extracellular electrical potential recordings. After detailing the different steps of the method, we assessed its performance using biophysical simulations. Afterwards, we validated our approach with experimental data recorded from two different HD-MEA devices - MEA1k and DualMode. We successfully reconstructed over 400 axonal branches and estimated the corresponding AP propagation velocities in two recording datasets. The developed algorithm and method can be used with all commercially available CMOS-based HD-MEAs. Moreover, we provide an open-source Python package available on GitHub ( https://github.com/alejoe91/axon_velocity ) and on PyPi ( https://pypi.org/project/axon-velocity/ ) to facilitate the adoption of the method.

### Comparison with previous work

The presented algorithm builds upon previous approaches to automatically reconstruct axonal arbors from HD-MEA extracellular signals. In *Yuan et al. 2020* [20], the authors introduced an axon-reconstruction method developed for the DualMode device. A basic idea of this approach that we also utilized for the method presented here, is to start the axon reconstruction *backwards*, i.e., from electrodes featuring late signal peak occurrences, which are most likely at the end of the respective axonal branches. However, a main limitation of this approach is that the search for axonal paths is *local*, i.e., that, in each step, the algorithm selects the next channel in the path only based on local signal amplitudes under the condition that the signal peak occurrence is earlier. This local search can result in zig-zag paths, as the algorithm has no information on the global structure of the signal landscape. To overcome this limitation, *Ronchi et al. 2020* [34] introduced a very first version of a graph-based algorithm. From there, we made several improvements that were facilitated by the model-based validation that we present here. First, we extended the list of available filters for channel selection; in [34] only detection and kurtosis filters were used; second, we changed the interrogation of the graph to find axonal branches from using only the distance criterion, i.e., shortest distance (which could result in shortcuts and undetected axonal segments) to using a combination of distance and amplitude (*h_edge_*) criteria with the A^*^ method; third, we changed the strategy to avoid duplicates in the path: instead of looking for and removing duplicate paths *a-posteriori*, we here utilized the set of neighboring channels to existing paths to avoid finding duplicates *a-priori*, which also resulted in a more efficient implementation. Finally, we added pruning, merging, and splitting steps that were not implemented in [34], which arguably provide a better estimation of the axon branches.

### Limitations

While the proposed method is, to the best of our knowledge, the first attempt of axonal tracing using HD-MEA signals in a fully-automated way and at high throughput, some limitations remain. Given the two-dimensional geometry of the recording electrode array, the method can only capture features in 2D and ignores modulations in the third dimension. A modulation in the z-distance of an axon to the MEA surface will result in a distorted estimate of axonal AP propagation velocity, as the distance traveled by the AP along a path in 3D will be different from its 2D projection onto the electrode plane. However, most neuronal preparations *in vitro* are 2D, at least most primary neuronal and organotypic cultures, where neurons and their neurites extend across a planar electrode array. Moreover, estimating the z-coordinate (the height above the electrode plane) in addition to the x-y coordinates of an axon is a complicated inverse problem. While the amplitude of the recorded axonal signal is known to depend on the position relative to the recording electrode, various other biophysical factors, such as ion-channel densities and kinetics, membrane capacitances, axial resistances, and axon geometries, can influence axonal AP conduction velocities in unmyelinated axons [45, 46, 47, 48, 49, 50]. In order to use the signal amplitude to correct for z-modulation, one would need to make assumptions about these other biophysical factors. A modulation of the z-distance could also result in undetectable axonal segments, for example, due to an axon passing over a glia cell or another neuron along its way. This z-distance change could result in a *jump* in the peak latency. A similar situation could occur when axonal branches cross each other. Clearly, such a situation represents a complication for any method attempting to track single axonal branches and it might also cause a *jump* from one branch to another. While we have not explicitly addressed the problem of undetected segments or crossing axons, our path splitting procedure should be able to cope with these issues, since it tries to split branches with jumps in latency and considers them as separate axonal branches.

Another potential caveat is that our method does not distinguish between axonal propagation and propagation through other neurites, like dendrites. While we currently do not identify and remove dendritic branches that might be reconstructed by the method, one could consider the lower propagation velocity of dendrites in comparison to axons and remove low-velocity branches. Additionally, extracellular signals in proximity to dendritic sections usually exhibit a different waveform (positive peaks) than axonal signals mainly as a consequence of capacitive return currents [51]. Analyzing branch-specific waveform features, such as the peak-to-trough ratio, could therefore provide indications to identify and potentially exclude dendrites from the reconstruction. While this is a limitation for axon tracking, it could also be considered a feature in case that one would want to also reconstruct and characterize dendritic back-propagation and its velocity.

The proposed method relies on spike sorting to obtain clean extracellular footprints that are used as input for the algorithm. Due to the high channel count of the HD-MEA devices that we used, we relied on automatic spike sorting methods to isolate units. However, these methods are known to have limitations [40], especially in regimes of highly synchronized firing activity (e.g., network bursts) [52], which are rather frequent in *in vitro* cell culture preparations [53]. Therefore, before running the axon reconstruction algorithm, we performed extensive manual curation to improve the spike sorting output, by removing noisy units and merging oversplit units (based on template similarity and cross-correlogram features). It is strongly advisable to carefully check the spike sorting output before using the axon tracking method to limit the extent of spike sorting errors that could distort the reconstruction results.

The method includes many parameters that have been carefully tuned to perform well on data of unmyelinated axons obtained with high-density MEAs (center-to-center-electrode distances of ∼ 17.5 μm). While the same approach can be applied with other designs and lower electrode densities, the reconstruction performance quickly degrades as the spatial resolution of the MEA decreases (Figure 6). However, the user could then modify some of the parameters to adapt the algorithm for use with different electrode densities. Most of the parameters have a physical meaning that should facilitate the choice of reasonable values. As an example, the peak_std_distance determines the radius in μm to select neighboring channels and compute the peak time standard deviation (Section 2.2.1) for a given channel selection: the default value is set to 30 μm, which should be certainly increased for using a MEA design with, for example, 50 μm inter-electrode distance (otherwise no neighbors would ever be selected). Nevertheless, the default parameters should provide a good first guess for data from commercially available HD-MEA devices (e.g. from Maxwell Byosystems - www.mxwbio.com , 3Brain - www.3brain.com , or Multichannel Systems - www.multichannelsystems.com ).

Finally, the validation of the proposed method was performed on simulated ground-truth data only. Experimental validation is still an essential step to further assess the performance of the method (with respect to different neuronal types, cell densities etc.) and to improve it for different use cases. A possible approach would include to use live imaging methods to identify axons in sparse cultures, for example calcein AM live-staining [54] or other fluorescence indicators to avoid shrinkage of the tissue due to fixation (required for standard staining procedures). As usually a comparably high density of neurons in the cell cultures (tens of thousands of neurons on the array) is required to establish stable networks with sustained activity, however, we anticipate that live staining methods, according to our experience, will yield very crowded images, which would make it very difficult to segment and identify individual axons and to unambiguously match electrically recorded and imaged neurons and axons: at each and any of the electrodes, there are plenty of visible neuronal structures that could have caused or contributed to a signal, and sometimes the structures generating the signals are not even visible, e.g., lying below other neurons or processes. Most probably, patch-clamping or functional calcium/voltage imaging, performed simultaneously with HD-MEA measurements, will enable an unambiguous correlation of extracellular neuronal recordings with neuronal and axonal morphology, as intra- and extracellular signals can be simultaneously triggered and recorded. We believe that such multi-modal approaches, despite being challenging to implement and to perform measurements, will enable a more thorough validation of the developed tracking algorithm, and we plan to use such methods in future studies.

### Applications to neurological disease characterization and network dynamics

An automated and sufficiently accurate method to estimate axonal AP propagation velocities from HD-MEA recordings holds great promise to study axonal electrophysiology and pathophysiological conditions related to axonal dysfunction. A panoply of pathological conditions impair axonal functions and mostly result in conduction delays, which ultimately may cause conduction failures [55, 56, 57, 58, 59, 60]. Axonal dysfunction due to demyelination (e.g., multiple sclerosis) [61, 62], acute axonal damage [63], and channelopathies, among others, are shown to change axonal AP conduction properties [64, 65, 66].

Axonal features, such as differences in axon growth, axon signal conduction, time-course of axon degeneration or axon excitability can also be included in electrophysiological phenotypic characterization of human induced pluripotent stem cell (hiPSC)-derived neuronal cultures. Such cultures are available from patients suffering from neurological disorders and from healthy donors, so that electrophysiological biomarkers associated to neurological diseases can be established. *Ronchi et al.* [34], for example, made a first attempt to characterize axonal velocities of hiPSC-derived neuronal cultures and found significant differences between healthy motor and dopaminergic neurons and disease phenotypes featuring mutations related to Amyotrophic Lateral Sclerosis (ALS) and Parkinson’s disease (PD).

Besides identification and characterization of neurological diseases, an accurate determination of axonal AP propagation velocity opens up pathways to investigate axonal conduction times and delays and their role in neuronal coding and plasticity. Repetitive activity can alter the excitability of axonal membranes and AP conduction velocity, which can result in substantial changes in AP timings and spike propagation to presynaptic sites [67, 2, 68]. Conduction delays, which depend on conduction velocity and axonal length, can vary during repetitive activity, resulting in altered spike timings and intervals. Such changes in temporal spike patterns may be an important feature in shaping the neural code [69, 70, 71]. Similarly, axonal conduction velocities are highly adaptive in neuronal circuits and undergo changes in unmyelinated axons upon depolarization or during formation of new myelin sheaths depending on neuronal activity [72, 73, 74]. Our algorithm helps to facilitate the study of axonal conduction and potential failures, as it enables to simultaneously track a larger number of different axonal branches, to assess AP propagation velocities and conduction delays and to study the role of plasticity of conduction velocity in network-level dynamics. Such applications can also be extended to various model preparations such as organotypic cultures, acute brain slices and retinal slices.

### Outlook

In conclusion, in this article, we introduced and validated a novel automated method for axonal reconstruction from HD-MEA recordings, which enables to track changes in axonal conduction velocity over days. By providing an open-source Python package to use and apply the algorithm, we envision rapid adoption by the electrophysiology and HD-MEA community, which will eventually boost our understanding of biophysical and computational properties of axons in healthy and diseased states.

## Supplementary Material

Appendix

## Figures and Tables

**Table 1:** Performance on simulated data of model cells. Each entry of the table reports the Cell model (1, 2, 3, 4), the branch IDs (corresponding to Figure 5), the ground-truth and estimated velocities, the absolute velocity error (in *mm/s*) and the relative error (in %). The last column displays the mean and standard deviation of the tracking error in μm.

model ID	branch ID	velocity GT (*mm/s*)	velocity est. (*mm/s*)	abs. vel. error (*mm/s*)	rel. vel. error (%)	tracking error (μm)
Cell 1	0	218	219	1	0.5	8.8±13.6
	1	224	220	4	1.6	7.7±7.2
	2	246	235	11	4.2	50.3±56.6
Cell 2	0	287	216	71	24.7	12.5±9.6
	1	260	225	35	13.6	7.3±3.7
	2	278	250	28	10.2	18.2±26.9
	3	279	256	23	8.2	12.5±8.1
	4	257	259	2	0.8	8.7±4.2
Cell 3	0	223	213	10	4.7	8.7±8.6
	1	221	223	2	0.7	11.3±23.5
	2	218	216	2	0.8	37.4±37.8
	3	220	151	69	31.5	29.9±42.4
	4	241	215	26	10.9	15.1±13.4
Cell 4	0	210	209	1	0.5	9.9±13.1
	1	258	250	8	2.9	14.5±9.9
	2	216	225	9	3.9	6.4±2.5
	3	194	181	13	6.7	20.6±29.1
	4	220	210	10	4.7	64.5±79.9
	5	218	219	1	0.4	4.8±2.0
	6	163	175	12	7.3	48.8±45.3
	7	235	195	40	16.8	77.5±84.2
	8	215	205	10	4.7	16.0±27.0
	9	222	204	18	7.9	12.9±19.6
	10	222	160	62	27.8	20.6±23.7
	11	216	214	2	0.8	13.8±18.8
	12	220	201	19	8.8	68.9±87.0
