# Supplementary material for: An automated method for precise axon reconstruction from recordings of high-density micro-electrode arrays
Source: J Neural Eng. Author manuscript; Available in PMC 2022 Apr 4. (PMC7612575; doi:10.1088/1741-2552/ac59a2)
Supplement: Appendix [file EMS143887-supplement-Appendix.pdf]

APPENDIX

A Raw axonal branch estimation algorithm

In this appendix, we report the pseudo-code for the algorithm to estimate raw axonal branches from the constructed graph (see Section 2.2.3). Note that the pruning and merging steps are not included.

```
input : graph, min_points, min_length, init_channel
output: raw_paths, branching_points
// list with raw paths
raw_paths = list()
// list with removed nodes per path
removed_neighbors_per_path = list()
// set with all removed nodes
all_removed_neighbors = set()
// list with branching points
branching_points = list()
// path indexes
path_idxs = list()
current_idx = 0
for source_node in graph.nodes do
    if source_node not in all_removed_neighbors then
        if is_local_maximum(source_node) then
            // Find shortest path to init channel
            path = astar_path(source_node, init_channel)
            // Remove nodes already in other paths and connect to branching point
            for i in path_idxs do
                removed_nodes_in_path = removed_neighbors_per_path(i)
                for node in path do
                    if node in removed_nodes_in_path then
                        // Remove further nodes along the path
                        path.remove(node:end)
                        // Find and append branching point
                        closest_node = find_closest_node(node, raw_paths(i))
                        path.append(closest_node)
                        possible_branching_point = closest_node
                    end
                end
            end
            if length(path) ≥ min_points and length_in_μm(path) > min_length then
                // Accept raw path
                raw_paths.append(path)
                // Update list of removed nodes
                neighbor_nodes = find_neighbors(path)
                removed_neighbors_per_path.append(neighbor_nodes)
                all_removed_neighbors = all_removed_neighbors ∪ neighbor_nodes
                branching_points.append(possible_branching_point)
                path_idxs.append(current_idx)
                current_idx += 1
            end
        end
    end
end
```

Algorithm 1: Identification of raw axonal paths from the graph.

Accepted Manuscript

B Description of parameters

In this appendix, we report a complete list of the parameters available for `axon_velocity` version 0.1.1. The parameters are listed in Table 2.

Parameter	Value	Type	Description
**General**			
upsample	1	int	upsampling factor for template
min_selected_points	30	int	minimum number of selected points to run axon tracking
verbose	False	bool	if True, the output is verbose
**Channel selection**			
detect_threshold	0.02	float	detection threshold (with respect to channel featuring maximal signal) below which a channel is discarded
detection_type	"relative"	string	whether to use an "absolute" or "relative" detection threshold
kurt_threshold	0.3	float	kurtosis threshold below which a channel is discarded
peak_std_threshold	1	float	peak time standard deviation threshold in ms below which a channel is discarded
init_delay	0.1	float	initial delay in seconds (with respect to maximum channel) below which a channel is discarded
peak_std_distance	30	float	distance in µm to select channel neighborhood to compute peak time standard deviation
remove_isolated	True	bool	if True, isolated channels are removed from selection
**Graph**			
init_amp_peak_ratio	0.2	float	scalar value that weighs the contribution of the amplitude and the peak latency for h_{init} (α_{init} in Eq. 2)
max_distance_for_edge	100	float	maximum distance in µm between channels to create a graph edge
max_distance_to_init	200	float	maximum distance in µm between a channel and the *init_channel* to create a graph edge below which an axonal branch is discarded
n_neighbors	3	int	maximum number of edges that one channel can connect to
distance_exp	2	float	exponent for distance computation (e in Eq 3)
edge_dist_amp_ratio	0.3	float	relative weight between distance and amplitude to select neighbor nodes for graph edges
**Axonal reconstruction**			
min_path_length	100	float	minimum axon path length in µm to include an axonal branch
min_path_points	5	int	minimum number of channels in an axon path to include an axonal branch
neighbor_radius	100	float	radius in µm to exclude neighboring channels around an identified path
min_points_after_branching	3	int	minimum number of points after a branching to avoid pruning
**Path cleaning/Velocity estimation**			
mad_threshold	8	float	threshold in median absolute deviations on the fit error to consider points as outliers in the velocity estimation
split_paths	True	bool	If True, the final path splitting step is enabled
max_peak_latency_for_splitting	0.5	float	If a *jump* in the peak latencies of a path exceeds this value, the path can be split in sub-paths
r2_threshold	0.9	float	R^2 threshold for velocity linear fit below which an axon branch is discarded
r2_threshold_for_outliers	0.98	float	R^2 threshold below which outliers are detected and removed
min_outlier_tracking_error	50	float	tracking error in µm above which a point can be considered an outlier and removed

Table 2: Additional parameters list for the `compute_graph_propagation_velocity()` function, including default values, data types, and descriptions.

Accepted Manuscript
